# Simulation of the influence of oxygen on the chemical stage of radiobiological mechanism using Petri nets

**DOI:** 10.1186/1758-2946-6-S1-P12

**Published:** 2014-03-11

**Authors:** J Barilla, M Lokajíček, H Pisaková, P Simr

**Affiliations:** 1 J. E. Purkinje University in Usti nad Labem, Faculty of Science, Czech Republic; 2 Institute of Physics, Academy of Sciences of the Czech Republic

## 

The radiobiological effect of densely ionizing ends of primary or secondary charged particles may be influenced significantly by processes running in the chemical stage of radiobiological mechanism; especially the influence of present oxygen may be very important. The effect of its or of other species (radiomodifiers) present in water medium during irradiation may be studied with the help of corresponding mathematical models. The model based on the use of Petri nets will be proposed and described.

Two parallel processes, i.e., diffusion of radicals and their chemical reactions, running in corresponding radical clusters formed during energy transfer may be represented with the help of the given model. A great number of chemical species may be easily taken into account. The model enables to study the concentrations of individual radicals changing during cluster diffusion and to estimate their damaging effects on corresponding DNA molecules in given cells. The results demonstrating the influence of oxygen under different concentrations will be presented.

